# Time to Exudative Recurrence After Complete Fluid Resolution Following Anti-VEGF Loading in Neovascular Age-Related Macular Degeneration

**DOI:** 10.3390/jcm15176835

**Published:** 2026-09-03

**Authors:** Kyungyun Kook, Dohee Park, GeonIl Lee, Yong-Sok Ji

**Affiliations:** 1 Department of Ophthalmology, Chonnam National University Medical School and Hospital, Gwangju 61469, Republic of Korea; ruddbs16@gmail.com (K.K.); dohesmanx@naver.com (D.P.); ygeonil@naver.com (G.L.); 2 Parangsae Eye Clinic, MSC Research Institute, Gwangju 61486, Republic of Korea

**Keywords:** neovascular age-related macular degeneration, anti-VEGF, exudative recurrence, fibrovascular pigment epithelial detachment, pro re nata, survival analysis

## Abstract

**Background/Objectives:** The timing of exudative recurrence after complete fluid resolution following anti-vascular endothelial growth factor (anti-VEGF) loading in neovascular age-related macular degeneration (nAMD) is unpredictable. We prespecified two questions: whether the speed of fluid resolution during loading, or the baseline pigment epithelial detachment (PED) type, predict recurrence timing. **Methods:** We retrospectively analyzed 72 treatment-naïve nAMD eyes that achieved complete resolution of subretinal fluid (SRF) and intraretinal fluid (IRF) after three monthly loading injections, then observed them under a pro re nata strategy. Time from the post-loading dry-confirmation visit to first detected recurrence was analyzed with Kaplan–Meier and Cox models; baseline variables with univariable *p* < 0.10 were entered into a multivariable model. **Results:** Recurrence was detected in 58 eyes (80.6%); median recurrence-free survival was 139 days (95% CI, 118–158). Resolution of SRF or IRF by one month was not significantly associated with recurrence timing (*p* = 0.586 and 0.054). Fibrovascular PED was associated with earlier detected recurrence compared with non-fibrovascular PED (adjusted hazard ratio 2.40, 95% CI, 1.17–4.92; *p* = 0.017; median 130 vs. 602 days). Eyes with fibrovascular PED were monitored more frequently, but the association persisted in an interval-censored sensitivity analysis (adjusted HR 2.71, 95% CI, 1.32–5.60). **Conclusions:** The speed of fluid resolution was not significantly associated with recurrence timing, whereas fibrovascular PED was associated with earlier detected recurrence and may represent a candidate prognostic marker requiring prospective validation.

## 1. Introduction

Neovascular age-related macular degeneration (nAMD) is a leading cause of irreversible vision loss in older adults, and its global burden is projected to increase with population aging [1]. Anti-VEGF therapy is the standard of care. Pivotal trials established that fixed monthly dosing provides substantial visual gains [2,3], but the treatment burden is considerable. Pro re nata (PRN) regimens, typically beginning with three monthly loading doses followed by re-treatment guided by disease activity [4,5], reduce injections but can lead to undertreatment when monitoring is insufficient [6,7,8].

Treat-and-extend (T&E) regimens reduce the risk of uncontrolled exudative recurrence through proactive dosing but increase cumulative treatment burden, and may expose some durably quiescent eyes to injections that would not have been required under observation [9]; more frequent dosing has also been associated with a higher risk of macular atrophy [10]. A minority of eyes remain quiescent after loading alone—about one in five required no further injections during the 9-month pro re nata phase of a flexible-dosing trial [5], and roughly 15% remained free of recurrence over 24 months in a PRN cohort [11]—whereas others recur soon after resolution, even under T&E [12,13]. Identifying, at the end of loading, which eyes are more likely to recur early could therefore help individualize monitoring.

Two candidate markers are available at that time point. First, the speed of fluid resolution during the loading phase is an intuitive indicator of treatment responsiveness and can be assessed as early as one month after the first injection; whether a rapid response translates into a longer quiescent interval, however, has not been established. Second, baseline morphology may matter independently of treatment response: fibrovascular PED reflects active neovascular tissue beneath the retinal pigment epithelium (RPE), and its absence has been associated with freedom from recurrence [11]. Macular neovascularization (MNV) is classified by anatomical location into types 1–3 and aneurysmal type 1/polypoidal choroidal vasculopathy (PCV) [14], and PCV has been reported to reactivate at shorter intervals [15]. Deep-learning models using early post-treatment optical coherence tomography (OCT) images have also been developed to predict recurrence, highlighting the difficulty of predicting recurrence using routine clinical assessment alone [16,17].

Fibrovascular PED has previously been associated with recurrence risk [11], and our aim was not to re-establish this marker itself. Rather, most prior studies dichotomized recurrence at a fixed time point, whereas the timing of recurrence after confirmed post-loading dryness has been less well characterized. We therefore analyzed the time to first detected recurrence, anchored at the post-loading dry-confirmation visit, in eyes subsequently managed with observation/PRN. Within this framework, we examined two prespecified exposures: the speed of fluid resolution during loading—specifically, the resolution of subretinal fluid (SRF) and intraretinal fluid (IRF) by one month—and baseline PED type. The principal contribution is thus the analysis of recurrence timing and of fluid-resolution speed after complete dryness, rather than the identification of fibrovascular PED as a risk marker.

## 2. Materials and Methods

### 2.1. Study Design and Participants

This retrospective cohort study was conducted at a single tertiary center. Treatment-naïve eyes with nAMD—defined by active MNV on OCT, fluorescein angiography (FA), and indocyanine green angiography (ICGA) with no prior anti-VEGF, photodynamic therapy, or laser treatment—that received three monthly loading injections of an anti-VEGF agent (aflibercept 2.0 mg [Eylea®, Bayer AG, Leverkusen, Germany] or ranibizumab 0.5 mg [Lucentis®, Norvatis Pharma AG, Basel, Switzerland]; no agent switching) between January 2018 and December 2024 were screened. Eligible eyes had complete resolution of subretinal and intraretinal fluid on OCT one month after the third loading injection. In patients with bilateral nAMD, each eye was assessed for eligibility independently. The eye was the unit of analysis.

Exclusion criteria were subfoveal fibrosis, atrophy, or scar; diabetic retinopathy; retinal vein occlusion; pathologic myopia; inflammatory or uveitic disease; intraocular surgery within three months before the first loading injection; and severe epiretinal membrane causing macular distortion. Eyes that did not achieve complete fluid resolution after loading, and eyes subsequently managed with a proactive T&E regimen, were not analyzed.

### 2.2. Imaging, Definitions, and Grading

OCT was performed with a spectral-domain device (Spectralis OCT, Heidelberg Engineering, Heidelberg, Germany) using enhanced depth imaging (EDI) mode. OCT images were analyzed using Heidelberg Eye Explorer software (HRA/SPECTRALIS Viewing Module, version 6.12.4.0; Heidelberg Engineering, Heidelberg, Germany). A 49-line horizontal raster covering 30° × 20° (8.9 × 5.9 mm) with 123 µm spacing between B-scans was acquired, with automatic real-time (ART) averaging of 12 frames per B-scan. Best-corrected visual acuity (BCVA) was measured with a Snellen chart and converted to the logarithm of the minimum angle of resolution (logMAR). Central retinal thickness (central 1 mm subfield) and subfoveal choroidal thickness (from the outer border of the RPE to the choroid–scleral interface beneath the fovea) were recorded. The MNV area was manually measured on ICGA images and expressed in mm^2^, and MNV was classified as type 1, type 2, type 3, or aneurysmal type 1/PCV using FA and ICGA, according to consensus nomenclature [14].

PED was defined as an RPE elevation exceeding 400 µm in width and 75 µm in height, or exceeding 200 µm in vertical height [18,19,20]. PED with a focal RPE elevation over an optically clear space was classified as serous; PED with moderately reflective material adherent beneath the RPE as fibrovascular; and PED over homogeneous drusenoid material as drusenoid. In ambiguous cases with mixed components, the dominant component was determined using corresponding FA/ICGA images. For analysis, eyes were grouped as fibrovascular PED versus non-fibrovascular PED (serous, drusenoid, or no PED). Two retina specialists independently graded PED type while masked to recurrence outcomes; disagreements were resolved by consensus. Pre-consensus agreement was 71 of 72 eyes (Cohen’s κ = 0.97).

Complete fluid resolution was defined as the absence of both subretinal and intraretinal fluid; persistent PED was not regarded as residual activity. Exudative recurrence was defined as the reappearance of intraretinal or subretinal fluid, or new subretinal hemorrhage, on OCT and/or fundus examination after complete resolution; a qualitative increase in PED was used only as a supportive criterion. The visit one month after the third loading injection, at which dryness was confirmed, served as the landmark (time zero). Recurrence was determined by the treating physician during routine clinical care. Subretinal and intraretinal fluid were graded independently by two retina specialists masked to recurrence outcomes, with complete agreement between the graders.

### 2.3. Variables and Outcomes

Baseline variables were age, sex, hypertension, diabetes, MNV subtype, anti-VEGF agent, MNV area, BCVA, central retinal thickness, subfoveal choroidal thickness, baseline SRF volume, presence of SRF and IRF, PED type (fibrovascular versus non-fibrovascular), and maximal PED height. SRF volume was manually quantified from serial OCT B-scans: the boundaries of SRF, defined as the hyporeflective space between the outer surface of the neurosensory retina and the RPE, were manually delineated on each B-scan containing SRF, the cross-sectional area was measured in square millimeters, and the total volume was obtained by numerical integration of the serial cross-sectional areas using the 123 µm interscan distance according to the Cavalieri principle, expressed in cubic millimeters. SRF volume was measured independently by two graders masked to clinical outcomes; intergrader reliability was assessed using a two-way random-effects, absolute-agreement, single-measure intraclass correlation coefficient (ICC = 0.909; 95% CI, 0.852–0.945) among eyes with baseline SRF.

Loading-phase response variables were the resolution of SRF and of IRF by one month (assessed among eyes with the corresponding fluid at baseline), the time to complete dry-up (1 versus 2–3 months), and changes in BCVA, central retinal thickness, and subfoveal choroidal thickness between baseline and month 3. After the dry-confirmation visit, eyes were monitored under observation/PRN at intervals determined by the treating physician, rather than by a standardized protocol, generally every 1–3 months, with earlier visits if symptoms developed; re-treatment was administered when exudative recurrence was detected. The choice between observation/PRN and treat-and-extend after loading was not determined by protocol or randomization, but was based on the treating physician’s clinical judgment and patient preference. The primary outcome was the time from the landmark to the first detected recurrence; eyes without detected recurrence were censored at the last fluid-free visit (median follow-up of censored eyes, 669 days; range, 155–1855).

### 2.4. Statistical Analysis

Continuous variables are summarized as mean ± standard deviation or median (interquartile range) as appropriate, and categorical variables as counts and percentages. Recurrence-free survival was estimated by the Kaplan–Meier method (with log–log 95% confidence intervals (CIs)) and compared between groups with the log-rank test; the median potential follow-up was estimated by the reverse Kaplan–Meier method. Associations with recurrence timing were assessed by univariable Cox proportional hazard regression for all baseline candidate variables. Variables with a univariable *p* < 0.10 were entered into a multivariable Cox model; this threshold was applied uniformly, and no other selection criterion was used. Hazard ratios (HRs) are reported with 95% CIs. The proportional-hazard assumption was examined using log-minus-log survival plots and a time-by-covariate interaction test. The prespecified loading-phase response variables were analyzed separately by univariable Cox regression, because they are measured after treatment and could lie on the causal pathway between baseline morphology and recurrence. Robustness of the principal finding was examined by (i) excluding eyes with drusenoid PED, (ii) adding the anti-VEGF agent, and (iii) adding MNV subtype as a four-level categorical variable.

For each eye, the median inter-visit interval and the total number of post-landmark visits were calculated from the post-loading dry-confirmation visit through the first detected recurrence visit or censoring. Eye-level median intervals and numbers of visits were compared between PED groups using the Mann–Whitney U test. Because recurrence was ascertained at follow-up visits and the exact time of recurrence therefore lay between the last recurrence-free visit and the first visit at which recurrence was detected, we additionally performed a sensitivity analysis, treating the outcome as interval-censored. For each eye that recurred, the interval was defined by the last dry visit and the first recurrence-positive visit; eyes without detected recurrence were right-censored at the last dry visit. A parametric proportional-hazard model with a Weibull baseline was fitted using the icenReg package (version 2.0.15) in R (version 4.3.1; R Foundation for Statistical Computing, Vienna, Austria), and hazard ratios were compared with those from the primary Cox analysis. All other analyses were performed using SPSS Statistics 21 (IBM Corp., Armonk, NY, USA).

## 3. Results

### 3.1. Study Population

**Figure 1:**
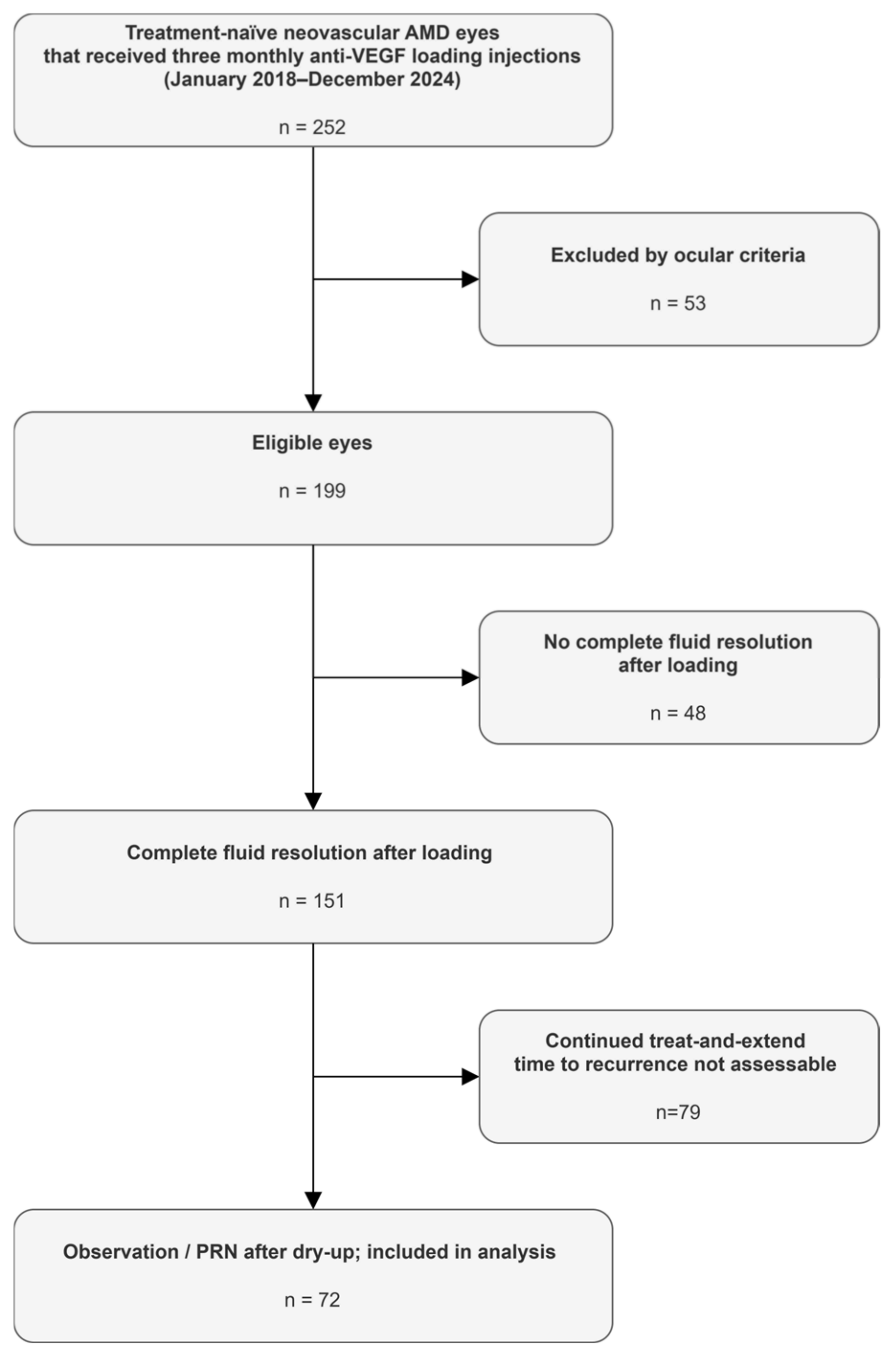
Flow of eyes through the study. Eyes managed with proactive treat-and-extend after loading were excluded because recurrence timing could not be assessed during an untreated observation interval. PRN, pro re nata.

Of 252 treatment-naïve nAMD eyes that received three monthly loading injections during the study period, 53 were excluded for prespecified ocular criteria and 48 did not achieve complete fluid resolution after loading. Of 151 eyes with complete resolution, 79 were subsequently managed with T&E—precluding observation-based assessment of recurrence timing—leaving 72 eyes managed by observation/PRN for analysis (Figure 1). Baseline characteristics are summarized in Table 1: mean age was 73.9 years, 47% were female, and diabetes was present in 28 patients (39%). MNV subtypes were type 1 in 12, type 2 in 21, type 3 in 26, and PCV in 13 eyes. On OCT, the baseline PED was fibrovascular in 53 eyes and non-fibrovascular in 19 eyes (serous, 4; drusenoid, 5; no PED, 10). No patient had both eyes meet all eligibility criteria, including post-loading management with observation/PRN; therefore, no patient contributed more than one eye.

We compared the 72 analyzed eyes with the 79 eyes that were dry after loading but subsequently managed with treat-and-extend (Table 2). Fibrovascular PED (74% vs. 76%; *p* = 0.886) and PCV (18% vs. 15%; *p* = 0.799) were similarly distributed between the groups, whereas the overall distribution of MNV subtypes differed (*p* = 0.002) with type 3 MNV more frequent in the observation/PRN group (36% vs. 11%) and type 1 and type 2 MNV more frequent in the treat-and-extend group (17% vs. 28% and 29% vs. 46%, respectively). The treat-and-extend group was also older, had poorer baseline BCVA, and more frequently had baseline SRF. These differences are consistent with non-random post-loading treatment allocation, and support the potential for selection bias.

### 3.2. Overall Recurrence

**Figure 2:**
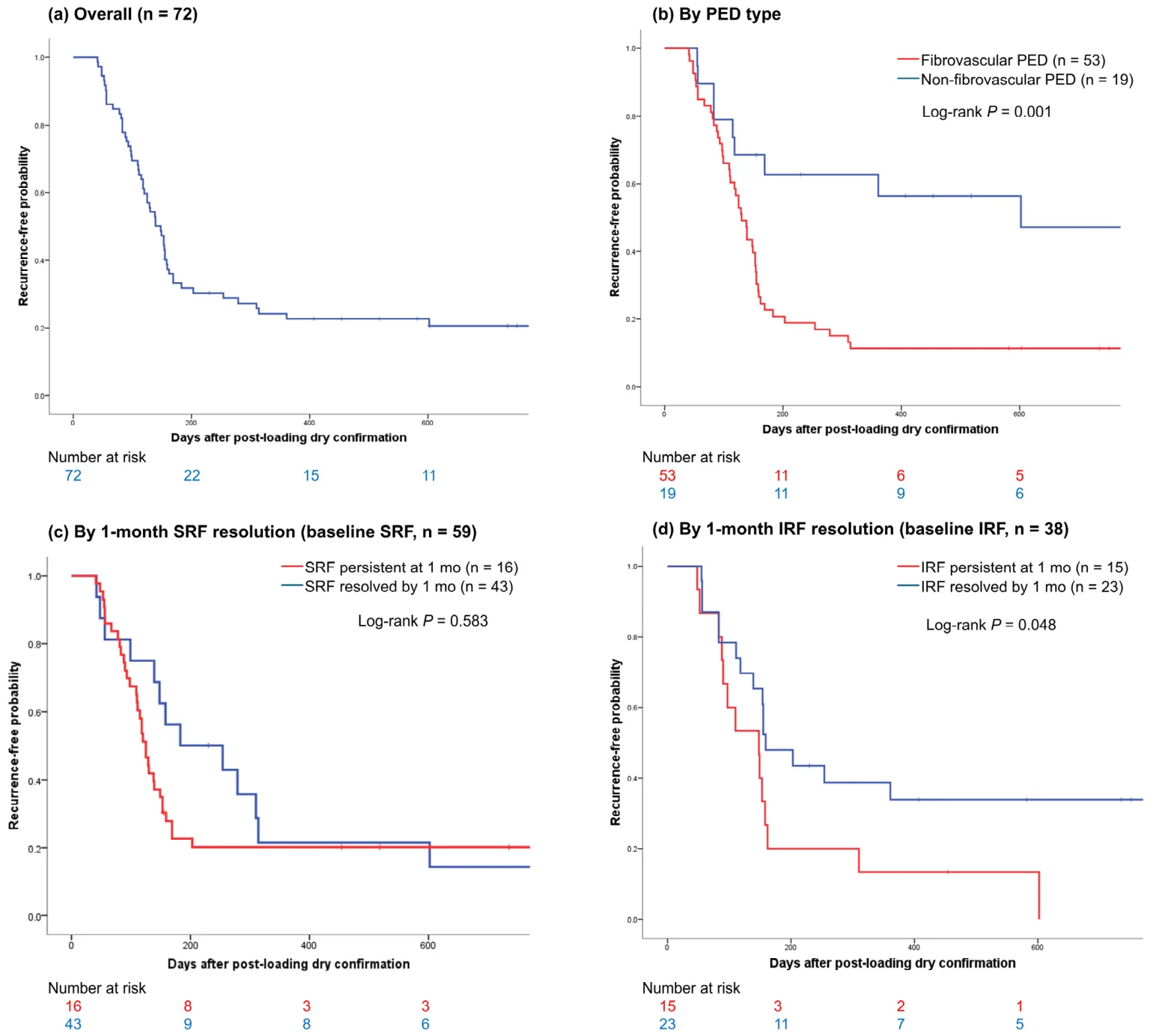
Kaplan–Meier recurrence-free survival from the post-loading dry-confirmation visit. (**a**) Overall cohort; (**b**) by baseline PED type; (**c**) by 1-month subretinal fluid (SRF) resolution among eyes with baseline SRF; (**d**) by 1-month intraretinal fluid (IRF) resolution among eyes with baseline IRF. Tick marks indicate censored eyes, and the number of eyes at risk at days 0, 200, 400, and 600 is shown below each panel. *p* values are from the log-rank test. Curves are displayed through 730 days for visual clarity; analyses used the complete follow-up period.

Exudative recurrence was detected in 58 eyes (80.6%); 14 eyes (19.4%) were censored without detected recurrence. The median potential follow-up (reverse Kaplan–Meier) was 751 days (24.7 months). The median recurrence-free survival was 139 days (95% CI, 118–158; Figure 2a). The estimated proportion remaining recurrence-free was 75.0% (95% CI, 63.3–83.4) at 3 months, 33.1% (22.5–44.0) at 6 months, and 22.7% (13.7–33.0) at 12 months; recurrence-free survival declined most steeply during the first 6 months.

### 3.3. Fibrovascular PED and Correlates of Recurrence Timing

Baseline characteristics stratified by PED type are shown in Table 3. Eyes with fibrovascular PED more often received aflibercept (83% vs. 47%; *p* = 0.006) and had greater maximal PED height (149 vs. 74 µm; *p* < 0.001). No other between-group differences reached statistical significance.

On univariable Cox analysis of all baseline candidate variables (Table 4; full list in Supplementary Table S1), fibrovascular PED was the only variable that reached statistical significance (HR 2.94, 95% CI, 1.46–5.90; *p* = 0.002). Baseline BCVA (*p* = 0.052) and diabetes (*p* = 0.082) met the prespecified *p* < 0.10 threshold for entry into the multivariable model, whereas PCV (*p* = 0.114), the anti-VEGF agent (*p* = 0.131), age, MNV area, and maximal PED height did not.

Eyes with fibrovascular PED had a median recurrence-free survival of 130 days (95% CI, 110–153) versus 602 days (118–771) for non-fibrovascular PED (48/53 versus 10/19 recurred; log-rank *p* = 0.001; Figure 2b). Descriptively, all three non-fibrovascular subgroups showed longer median recurrence-free survival than the fibrovascular group, although estimates were imprecise because of the small subgroup sizes (Supplementary Table S2). In the multivariable model containing fibrovascular PED, baseline BCVA, and diabetes, fibrovascular PED remained associated with earlier detected recurrence (adjusted HR 2.40, 95% CI, 1.17–4.92; *p* = 0.017), whereas baseline BCVA (HR 0.91 per 0.1 logMAR, 95% CI, 0.82–1.02; *p* = 0.108) and diabetes (HR 1.55, 95% CI, 0.89–2.72; *p* = 0.124) were not (Table 4). PCV showed substantial overlap with fibrovascular PED, with 12 of 13 PCV eyes (92%) classified as fibrovascular (Table 5). No evidence of violation of the proportional-hazard assumption was observed for any variable in the multivariable model (time-by-covariate interaction *p* = 0.90, 0.20, and 0.61 for fibrovascular PED, BCVA, and diabetes, respectively).

The overall median inter-visit interval was 42 days (IQR, 37–63), and the median number of post-landmark visits per eye was four (IQR, 3–7). Monitoring was more frequent in eyes with fibrovascular PED (median inter-visit interval, 42 days [IQR, 35–56] versus 63 days [IQR, 50–76]; *p* = 0.002), whereas the median number of post-landmark visits per eye did not differ significantly between groups (4 [IQR, 3–5] versus 5 [IQR, 3–10]; *p* = 0.220). To account for the difference in monitoring frequency and the resulting potential difference in detection timing, we repeated the analysis, treating recurrence as interval-censored. Fibrovascular PED remained associated with earlier recurrence in both univariable (Weibull HR 3.47, 95% CI 1.73–6.94; *p* < 0.001) and multivariable (adjusted HR 2.71, 95% CI 1.32–5.60; *p* = 0.007) interval-censored models, consistent with the primary Cox results (Supplementary Table S3).

### 3.4. Loading-Phase Response

None of the prespecified loading-phase response variables reached statistical significance in the primary Cox analyses (Table 6). Among eyes with baseline SRF (*n* = 59), resolution of SRF by one month was not associated with a longer recurrence-free interval; the point estimate was, if anything, in the direction opposite to that intuitively expected (median 125 versus 183 days for persistent SRF; HR 1.19, 95% CI, 0.64–2.19; *p* = 0.586; Figure 2c). Among eyes with baseline IRF (*n* = 38), resolution of IRF by one month showed a possible signal toward longer durability that did not reach significance in the primary Cox analysis (HR 0.48, 95% CI, 0.23–1.01; Cox *p* = 0.054; log-rank *p* = 0.048; Figure 2d); because the two groups had similar median survival (159 versus 148 days) and the curves separated only later in follow-up, this estimate should be interpreted with caution. The time to complete dry-up (2–3 versus 1 month) and loading-phase changes in BCVA, central retinal thickness, and subfoveal choroidal thickness were not associated with recurrence.

### 3.5. Robustness Analysis

The association between fibrovascular PED and earlier detected recurrence was consistent across additional robustness analyses (Supplementary Table S4). Excluding eyes with drusenoid PED, a non-exudative entity grouped with non-fibrovascular PED in the primary analysis, the adjusted HR was 2.73 (95% CI, 1.21–6.19; *p* = 0.016; *n* = 67). Adding MNV subtype (types 1, 2, 3, and PCV) to the multivariable model gave an adjusted HR of 2.28 (95% CI, 1.09–4.74; *p* = 0.028) for fibrovascular PED. PCV showed substantial overlap with fibrovascular PED (12 of 13 eyes; Table 5), limiting the ability to distinguish their independent associations. Adding the anti-VEGF agent gave an adjusted HR of 2.25 (95% CI, 1.10–4.59; *p* = 0.027), with the agent itself not associated (aflibercept versus ranibizumab, *p* = 0.419).

## 4. Discussion

Among nAMD eyes that achieved complete fluid resolution after three loading injections and were followed by observation/PRN, most had a detected recurrence within a few months (median, 139 days). Although a rapid fluid response might intuitively be expected to indicate greater durability, the speed of fluid resolution during loading was not significantly associated with recurrence timing in the primary Cox analysis. By contrast, fibrovascular PED was the only baseline variable significantly associated with earlier detected recurrence, and this association persisted after multivariable adjustment and across robustness analyses. These findings are exploratory and hypothesis-generating.

That an early, rapid response did not translate into a longer recurrence-free interval may seem clinically counterintuitive, but is understandable in the context of our selected cohort: by design, all included eyes ultimately achieved complete dryness, so what differed between eyes was how quickly they reached that state, rather than whether or not they did. Within such a cohort, characteristics of the underlying neovascular lesion—rather than the rate at which exudation cleared—may be more relevant to recurrence. The exploratory signal for IRF, but not SRF, resolution is broadly consistent with evidence that intraretinal fluid carries greater prognostic weight for visual outcomes than subretinal fluid [21,22]; however, whether this distinction also applies to recurrence timing remains uncertain. The borderline *p* value, small subgroup, and late separation of the curves warrant caution.

Consistent with our aim, the association between fibrovascular PED and recurrence was not itself a novel finding, but, rather, aligns with and extends prior work. Cho et al. found that absence of fibrovascular PED was associated with freedom from recurrence over 24 months [11], and fibrovascular PED has been linked to poorer treatment response in eyes with submacular hemorrhage [23]. Our data extend these observations to the timing of detected recurrence (adjusted HR 2.40, 95% CI, 1.17–4.92). The corresponding medians were 130 days (95% CI, 110–153) versus 602 days (118–771); the latter estimate is imprecise, reflecting the smaller non-fibrovascular group and its higher proportion of censored eyes, so the contrast in medians is illustrative, rather than a precise effect size. The association may reflect differences in the composition and activity of sub-RPE neovascular tissue between fibrovascular and non-fibrovascular PED, although the underlying mechanism cannot be determined from this study. Notably, maximal PED height was not associated with recurrence, suggesting that PED composition may be more informative than its size, although the null result for height does not exclude a smaller effect.

In our cohort, the Kaplan–Meier estimate of eyes remaining recurrence-free at 12 months was 22.7%. Direct comparison with prior cohorts is limited by differing follow-up durations and analytic time origins: Cho et al. reported that approximately 15% of eyes remained free of recurrence over a fixed 24-month PRN follow-up [11]. Because time to detected recurrence was measured from the post-loading dry-confirmation visit, rather than from the first injection [24], the intervals reported here begin approximately three months later in the treatment course, and are not directly comparable with those anchored at treatment initiation. Therefore, these recurrence-free estimates should be interpreted descriptively, rather than as direct evidence of higher or lower recurrence than in previous cohorts.

Clinically, complete dryness after loading did not guarantee durable quiescence, and recurrence timing remained difficult to predict from routinely available variables. A rapid loading-phase response alone, particularly the early clearance of subretinal fluid, should not be considered evidence of durable quiescence, whereas baseline PED type—readily identified on pretreatment OCT—may provide prognostic information. Given the small, selected cohort and the substantial overlap between fibrovascular PED and PCV, however, fibrovascular PED should be regarded as a candidate prognostic marker, rather than a criterion for setting follow-up interval, and requires confirmation in larger prospective studies before it can guide monitoring decisions. Because this cohort was managed with observation/PRN and did not compare treatment regimens, our data do not establish a benefit of proactive treatment; rather, these findings may help inform future studies of risk-stratified monitoring strategies. Finally, contemporary nAMD management increasingly relies on treat-and-extend regimens and longer-acting anti-VEGF agents, which may limit the direct applicability of these observation/PRN-based findings to current practice.

This study has limitations. It was retrospective, single-center, and modest in size, limiting power and the stability of multivariable estimates. The analysis plan was not prespecified in a registered protocol; although the two exposures and the covariate-entry rule are reported as applied, the findings should be regarded as exploratory, and require external validation. Recurrence was ascertained at follow-up visits scheduled at the treating physician’s discretion, generally every 1–3 months or earlier, for symptoms, and treating physicians were not masked to baseline morphology. Because the retrospective records did not allow scheduled and symptom-triggered visits to be reliably distinguished throughout follow-up, the frequency of symptom-triggered visits could not be compared between PED groups. Accordingly, the outcome represents the time to detected recurrence rather than the true time of recurrence, and is interval-censored. Eyes with fibrovascular PED were in fact monitored more frequently (median interval 42 versus 63 days), which could, in principle, lead to earlier detection in this group. To address this, we compared monitoring intervals between groups and performed an interval-censored sensitivity analysis anchored on each eye’s last dry and first recurrence-positive visits; fibrovascular PED remained associated with earlier recurrence (adjusted HR 2.71, 95% CI 1.32–5.60), suggesting that the association was not fully explained by differences in monitoring frequency. Nonetheless, the outcome remains the time to detected recurrence, and residual detection bias cannot be fully excluded. PED type was classified using established criteria with near-perfect agreement on independent grading (κ = 0.97), although classification of mixed PEDs can be difficult.

Two sources of selection bias apply. First, inclusion required complete post-loading dryness, conditioning on a favorable early response. Second, the choice between observation/PRN and treat-and-extend was made by the treating physician, rather than by randomization; the 79 eyes subsequently managed with treat-and-extend—potentially those judged clinically to be at higher risk—were excluded because recurrence timing cannot be assessed during proactive treatment. The analyzed cohort may therefore be selected toward a particular risk profile, and the absolute recurrence rates and the magnitude of the associations may not generalize to all eyes achieving dryness after loading. Finally, post-loading PED persistence and morphological change were not systematically quantified in the source dataset, and could not be reliably reconstructed retrospectively. Post-recurrence visual acuity, retreatment burden, and hemorrhagic outcomes were likewise not systematically collected as study variables, and therefore could not be reliably analyzed. Whether or not post-loading PED characteristics or the downstream consequences of earlier recurrence carry additional prognostic value remains to be determined in future studies.

## 5. Conclusions

Among treatment-naïve nAMD eyes achieving complete fluid resolution after three loading injections, most had a detected recurrence within a few months. The speed of fluid resolution during loading was not significantly associated with recurrence timing, whereas fibrovascular PED remained associated with earlier detected recurrence after multivariable adjustment. Fibrovascular PED may therefore represent a candidate prognostic marker for earlier detected recurrence, but its role in guiding follow-up intervals requires confirmation in larger prospective studies with standardized monitoring and interval-censored analyses.

## Figures and Tables

**Table 1:** Baseline characteristics (72 eyes).

Characteristic	Value
Age, years (mean ± SD)	73.9 ± 8.5
Female, *n* (%)	34 (47)
Hypertension, *n* (%)	40 (56)
Diabetes mellitus, *n* (%)	28 (39)
MNV subtype, *n* (type 1/2/3/PCV)	12/21/26/13
PED type, *n* (fibrovascular/non-fibrovascular)	53/19
Anti-VEGF agent, *n* (aflibercept/ranibizumab)	53/19
MNV area (ICGA), mm^2^, median (IQR)	0.35 (0.16–0.75)
Baseline BCVA, logMAR (mean ± SD)	0.33 ± 0.25
Central retinal thickness, µm (mean ± SD)	388 ± 110
Subfoveal choroidal thickness, µm (mean ± SD)	230 ± 110
Baseline SRF volume, mm^3^, median (IQR)	0.91 (0.05–3.59)
SRF present, *n* (%)	59 (82)
IRF present, *n* (%)	38 (53)
PED maximal height, µm, median (IQR)	138 (90–202)

Continuous variables are presented as mean ± SD or median (IQR). Non-fibrovascular PED comprised serous (*n* = 4), drusenoid (*n* = 5), or no PED (*n* = 10). BCVA, best-corrected visual acuity; ICGA, indocyanine green angiography; IQR, interquartile range; IRF, intraretinal fluid; MNV, macular neovascularization; PCV, polypoidal choroidal vasculopathy; PED, pigment epithelial detachment; SRF, subretinal fluid.

**Table 2:** Baseline characteristics of eyes managed with PRN versus eyes that achieved complete post-loading fluid resolution but were subsequently managed with treat-and-extend (excluded from the recurrence-timing analysis).

Characteristic	Observation/PRN (*n* = 72)	Treat-and-Extend (*n* = 79)	*p*
Age, years	74.0 (69.0–80.0)	80.0 (74.5–83.5)	<0.001
Female, *n* (%)	34 (47)	26 (33)	0.103
Hypertension, *n* (%)	40 (56)	43 (54)	1.000
Diabetes mellitus, *n* (%)	28 (39)	35 (44)	0.611
MNV subtype, *n* (type 1/2/3/PCV)	12/21/26/13	22/36/9/12	0.002
Fibrovascular PED, *n* (%)	53 (74)	60 (76)	0.886
PCV, *n* (%)	13 (18)	12 (15)	0.799
Aflibercept, *n* (%)	53 (74)	64 (81)	0.372
Baseline BCVA, logMAR	0.3 (0.2–0.5)	0.7 (0.4–0.8)	<0.001
SRF present, *n* (%)	59 (82)	79 (100)	<0.001
IRF present, *n* (%)	38 (53)	44 (56)	0.845

Continuous variables are presented as median (IQR) and categorical variables as n (%). *p* values were calculated using the Mann–Whitney U test for continuous variables and the χ^2^ test or Fisher’s exact test, as appropriate, for categorical variables. These comparisons are descriptive and are intended to characterize potential selection associated with post-loading management, rather than to compare treatment effects. BCVA, best-corrected visual acuity; IRF, intraretinal fluid; MNV, macular neovascularization; PCV, polypoidal choroidal vasculopathy; PED, pigment epithelial detachment; PRN, pro re nata; SRF, subretinal fluid.

**Table 3:** Baseline characteristics of the analyzed cohort (72 eyes) stratified by fibrovascular versus non-fibrovascular PED.

Variable	Fibrovascular (*n* = 53)	Non-Fibrovascular (*n* = 19)	*p*
Age, years	74.0 (69.0–80.0)	74.0 (68.5–79.0)	0.539
Female, *n* (%)	23 (43)	11 (58)	0.413
Hypertension, *n* (%)	29 (55)	11 (58)	1.000
Diabetes mellitus, *n* (%)	24 (45)	4 (21)	0.099
PCV, *n* (%)	12 (23)	1 (5)	0.162
Aflibercept, *n* (%)	44 (83)	9 (47)	0.006
BCVA, logMAR	0.3 (0.2–0.5)	0.4 (0.1–0.6)	0.634
CRT, µm	362 (302–456)	350 (294–457)	0.888
SFCT, µm	186 (141–306)	245 (174–280)	0.486
SRF present, *n* (%)	44 (83)	15 (79)	0.734
IRF present, *n* (%)	26 (49)	12 (63)	0.430
PED maximal height, µm	149 (106–223)	74 (66–103)	<0.001
MNV area, mm^2^	0.4 (0.2–0.9)	0.2 (0.1–0.5)	0.105

Continuous variables are presented as median (IQR) and were compared using the Mann–Whitney U test; categorical variables were compared using the χ^2^ test or Fisher’s exact test, as appropriate. Abbreviations as in Table 2.

**Table 4:** Univariable and multivariable Cox analysis of baseline variables for time to detected exudative recurrence.

Variable	Univariable HR (95% CI)	*p*	Multivariable HR (95% CI)	*p*
Fibrovascular PED (vs. non-fibrovascular)	2.94 (1.46–5.90)	0.002	2.40 (1.17–4.92)	0.017
Baseline BCVA (per 0.1 logMAR)	0.91 (0.82–1.00)	0.052	0.91 (0.82–1.02)	0.108
Diabetes mellitus	1.60 (0.94–2.73)	0.082	1.55 (0.89–2.72)	0.124
PCV (vs. other MNV)	1.65 (0.89–3.07)	0.114	—	—
Aflibercept (vs. ranibizumab)	1.61 (0.87–2.97)	0.131	—	—
Age (per year)	1.00 (0.97–1.02)	0.737	—	—
MNV area (per mm^2^)	0.99 (0.69–1.41)	0.948	—	—
PED maximal height (per 10 µm)	1.01 (0.99–1.03)	0.396	—	—

Variables with a univariable *p* < 0.10 were entered into the multivariable model. A full univariable analysis of all baseline variables is provided in Supplementary Table S1. Abbreviations as in Table 1; CI, confidence interval; HR, hazard ratio.

**Table 5:** Cross-tabulation of baseline PED type and MNV subtype in the analyzed cohort (72 eyes).

PED Type	Type 1	Type 2	Type 3	PCV	Total
Fibrovascular	9	14	18	12	53
Serous	0	1	3	0	4
Drusenoid	0	3	2	0	5
No PED	3	3	3	1	10
Total	12	21	26	13	72

Of the 13 PCV eyes, 12 (92%) had fibrovascular PED; this near-complete overlap between PCV and fibrovascular PED limits the ability to separate their independent associations with recurrence. MNV, macular neovascularization; PCV, polypoidal choroidal vasculopathy; PED, pigment epithelial detachment.

**Table 6:** Prespecified loading-phase response variables and time to detected recurrence: univariable Cox analysis.

Loading-Phase Variable	Eyes	HR (95% CI)	*p*
SRF resolution by 1 month (eyes with baseline SRF)	59	1.19 (0.64–2.19)	0.586
IRF resolution by 1 month (eyes with baseline IRF)	38	0.48 (0.23–1.01)	0.054
Dry-up at 2–3 vs. 1 month	72	1.11 (0.65–1.88)	0.711
Δ BCVA, per 0.1 logMAR (baseline to month 3)	72	1.00 (0.88–1.14)	0.982
Δ CRT, per 10 µm (baseline to month 3)	72	1.00 (0.98–1.02)	0.883
Δ SFCT, per 10 µm (baseline to month 3)	72	1.05 (0.98–1.13)	0.159

HR < 1 indicates longer recurrence-free survival. CRT, central retinal thickness; SFCT, subfoveal choroidal thickness; other abbreviations as in Table 1.

## Data Availability

The data presented in this study are available on request from the corresponding author. The data are not publicly available owing to privacy and institutional restrictions on patient information.

## Supplementary Materials

The following supporting information can be downloaded at: https://www.mdpi.com/article/10.3390/jcm15176835/s1, Table S1. Univariable Cox proportional-hazards analysis of all baseline variables for time to detected exudative recurrence (72 eyes, 58 events). Table S2. Recurrence by lesion subgroup in the analyzed cohort (72 eyes), showing the composition of the non-fibrovascular comparison group and the overlap between fibrovascular PED and PCV. Table S3. Monitoring intervals by PED type and interval-censored sensitivity analysis for the association between fibrovascular PED and recurrence. Table S4. Robustness analyses for the association between fibrovascular PED and time to detected exudative recurrence.

## Abbreviations

The following abbreviations are used in this manuscript:

ART	Automatic real-time
BCVA	Best-corrected visual acuity
EDI	Enhanced depth imaging
FA	Fluorescein angiography
ICGA	Indocyanine green angiography
IRF	Intraretinal fluid
logMAR	Logarithm of the minimum angle of resolution
MNV	Macular neovascularization
nAMD	Neovascular age-related macular degeneration
OCT	Optical coherence tomography
PCV	Polypoidal choroidal vasculopathy
PED	Pigment epithelial detachment
PRN	Pro re nata
RPE	Retinal pigment epithelium
SRF	Subretinal fluid
T&E	Treat-and-extend
VEGF	Vascular endothelial growth factor
