# Supplementary material for: Time to Exudative Recurrence After Complete Fluid Resolution Following Anti-VEGF Loading in Neovascular Age-Related Macular Degeneration
Source: J Clin Med. 2026 Sep 3;15(17):6835. doi: 10.3390/jcm15176835 (PMC13565891; doi:10.3390/jcm15176835)
Supplement: Supplementary file 1 [file jcm-15-06835-s001.zip › jcm-4471912-supplementary.pdf]

Supplementary Information

Time to Exudative Recurrence After Complete Fluid Resolution Following Anti-VEGF Loading in Neovascular Age-Related Macular Degeneration

Kyungyun Kook, Dohee Park, GeonIl Lee and Yong-Sok Ji

Supplementary Table S1. Univariable Cox proportional-hazards analysis of all baseline variables for time to detected exudative recurrence (72 eyes, 58 events).

Variable	HR (95% CI)	P
Age (per year)	1.00 (0.97–1.02)	0.737
Female sex	1.04 (0.62–1.75)	0.871
Hypertension	0.80 (0.48–1.36)	0.413
Diabetes mellitus	1.60 (0.94–2.73)	0.082
Fibrovascular PED (vs non-fibrovascular)	2.94 (1.46–5.90)	0.002
MNV type 1 (vs. others)	1.09 (0.55–2.17)	0.805
MNV type 2 (vs. others)	0.72 (0.40–1.30)	0.273
MNV type 3 (vs. others)	0.92 (0.54–1.58)	0.766
PCV (vs. other MNV)	1.65 (0.89–3.07)	0.114
Aflibercept (vs. ranibizumab)	1.61 (0.87–2.97)	0.131
MNV area (per mm^2)	0.99 (0.69–1.41)	0.948
Baseline BCVA (per 0.1 logMAR)	0.91 (0.82–1.00)	0.052
Central retinal thickness (per 10 μm)	1.00 (0.98–1.03)	0.751
Subfoveal choroidal thickness (per 10 μm)	1.00 (0.97–1.02)	0.700
Baseline SRF volume (per mm^3)	1.02 (0.97–1.06)	0.440
SRF present	1.44 (0.71–2.94)	0.316
IRF present	0.76 (0.45–1.27)	0.293
PED maximal height (per 10 μm)	1.01 (0.99–1.03)	0.396

BCVA, best-corrected visual acuity; CI, confidence interval; HR, hazard ratio; IRF, intraretinal fluid; MNV, macular neovascularization; PCV, polypoidal choroidal vasculopathy; PED, pigment epithelial detachment; SRF, subretinal fluid.

Supplementary Table S2. Recurrence by lesion subgroup in the analyzed cohort (72 eyes), showing the composition of the non-fibrovascular comparison group and the overlap between fibrovascular PED and PCV.

A. By PED subtype

PED subtype	Eyes, n	Recurred, n (%)	Median RFS, days	95% CI
Fibrovascular	53	48 (91)	130	110–153

Serous	4	2 (50)	361	—
Drusenoid	5	3 (60)	771	—
No PED	10	5 (50)	602	—

B. By PCV and fibrovascular PED status

Group	Eyes, n	Recurrences, n
Non-PCV, non-fibrovascular PED	18	9
Non-PCV, fibrovascular PED	41	36
PCV, non-fibrovascular PED	1	1
PCV, fibrovascular PED	12	12
**Total**	**72**	**58**

Panel A: median recurrence-free survival (RFS) and 95% CIs from the Kaplan–Meier method; CIs are not estimable (—) in the small non-fibrovascular subgroups. Descriptively, all three non-fibrovascular subgroups showed longer median RFS than the fibrovascular group, although estimates were imprecise because of the small subgroup sizes. Panel B: fibrovascular PED was present in 53 of 72 eyes (74%), including 12 of 13 PCV eyes; this near-complete overlap limits the ability to distinguish the independent associations of PCV and fibrovascular PED with recurrence. CI, confidence interval; PCV, polypoidal choroidal vasculopathy; PED, pigment epithelial detachment; RFS, recurrence-free survival.

Supplementary Table S3. Monitoring intervals by PED type and interval-censored sensitivity analysis for the association between fibrovascular PED and recurrence.
A. Inter-visit monitoring interval

Group	Median interval, days (IQR)	Post-landmark visits per eye, median (IQR)
Overall	42 (37–63)	4 (3–7)
Fibrovascular PED	42 (35–56)	4 (3–5)
Non-fibrovascular PED	63 (50–76)	5 (3–10)
P value	0.002	0.220

B. Interval-censored proportional-hazards model (Weibull baseline)

Variable	HR (95% CI)	P
Fibrovascular PED — univariable	3.47 (1.73–6.94)	<0.001
Fibrovascular PED — multivariable	2.71 (1.32–5.60)	0.007

Baseline BCVA (per 0.1 logMAR) — multivariable	0.89 (0.80–0.99)	0.039
Diabetes mellitus — multivariable	1.57 (0.88–2.81)	0.127

Panel A: For each eye, inter-visit intervals were calculated from the post-loading dry-confirmation visit through the first detected recurrence visit or censoring, and post-landmark visit counts included visits after the landmark through the first detected recurrence visit or censoring. Eye-level median inter-visit intervals and visit counts were compared between PED groups using the Mann–Whitney U test. Panel B: recurrence was treated as interval-censored between each eye's last recurrence-free visit and first recurrence-positive visit, using a parametric proportional-hazards model with a Weibull baseline; the multivariable model contained fibrovascular PED, baseline BCVA, and diabetes, matching the primary Cox model. Fibrovascular PED remained associated with earlier recurrence, indicating that the association persisted after accounting for interval censoring, although residual bias related to informative visit scheduling cannot be excluded. BCVA, best-corrected visual acuity; CI, confidence interval; HR, hazard ratio; IQR, interquartile range; PED, pigment epithelial detachment.

Supplementary Table S4. Robustness analyses for the association between fibrovascular PED and time to detected exudative recurrence.

**Analysis**	**Fibrovascular PED HR (95% CI)**	**P**
Primary multivariable model (fibrovascular PED + BCVA + diabetes)	2.40 (1.17–4.92)	0.017
Excluding eyes with drusenoid PED (n = 67)	2.73 (1.21–6.19)	0.016
Adding MNV subtype	2.28 (1.09–4.74)	0.028
Adding anti-VEGF agent to the multivariable model	2.25 (1.10–4.59)	0.027

Each row reports the hazard ratio for fibrovascular versus non-fibrovascular PED under a different specification. Drusenoid *PED, a non-exudative entity, was grouped with non-fibrovascular PED in the primary analysis. When MNV subtype was added as a four-level categorical covariate, the association between fibrovascular PED and recurrence remained similar. PCV eyes were predominantly fibrovascular (12 of 13), limiting separation of their independent associations. The anti-VEGF agent was likewise not associated (aflibercept versus ranibizumab, HR 1.33; P = 0.419). BCVA, best-corrected visual acuity; CI, confidence interval; HR, hazard ratio; MNV, macular neovascularization; PCV, polypoidal choroidal vasculopathy; PED, pigment epithelial detachment.*
